# Construction of *Mycoplasma hyopneumoniae* P97 Null Mutants

**DOI:** 10.3389/fmicb.2021.518791

**Published:** 2021-04-22

**Authors:** Jeannett M. Clampitt, Melissa L. Madsen, F. Chris Minion

**Affiliations:** Department of Veterinary Microbiology and Preventive Medicine, Iowa State University, Ames, IA, United States

**Keywords:** *Mycoplasma hyopneumoniae*, adherence protein, null mutant, immunoblot, adherence assay, P97, recombination

## Abstract

*Mycoplasma hyopneumoniae* is the causative agent of enzootic pneumonia, a world-wide problem in the pig industry. This disease is characterized by a dry, non-productive cough, labored breathing, and pneumonia. Despite years of research, vaccines are marginally effective, and none fully protect pigs in a production environment. A better understanding of the host-pathogen interactions of the *M. hyopneumoniae*-pig disease, which are complex and involve both host and pathogen components, is required. Among the surface proteins involved in virulence are members of two gene families called P97 and P102. These proteins are the adhesins directing attachment of the organism to the swine respiratory epithelium. P97 is the major ciliary binding adhesin and has been studied extensively. Monoclonal antibodies that block its binding to swine cilia have contributed extensively to its characterization. In this study we use recombination to construct null mutants of P97 in *M. hyopneumoniae* and characterize the resulting mutants in terms of loss of protein by immunoblot using monoclonal antibodies, ability to bind purified swine cilia, and adherence to PK15 cells. Various approaches to recombination with this fastidious mycoplasma were tested including intact plasmid DNA, single-stranded DNA, and linear DNA with and without a heterologous RecA protein. Our results indicate that recombination can be used to generate site-specific mutants in *M. hyopneumoniae.* P97 mutants are deficient in cilia binding and PK15 cell adherence, and lack the characteristic banding pattern seen in immunoblots developed with the anti-P97 monoclonal antibody.

## Introduction

*Mycoplasma* constitute a genus of bacteria that cause disease in humans, fish, plants, and animals. They are unusual in that they lack cell walls and have a diminutive genome due to degenerative evolution. *Mycoplasma hyopneumoniae* is a highly ubiquitous and contagious bacterium that causes chronic porcine enzootic pneumonia (Thacker and Minion, 2012). It has been a global issue in the porcine industry for years, contributing 200 million to 1 billion dollars of economic losses per annum to the swine industry. Infection is spread by direct contact with nasal secretions or aerosol transmission from infected pigs. Hallmarks of infection are a dry, non-productive cough usually 10–14 days post infection and epithelial cell death in the trachea, bronchi, and bronchioles (Debey et al., 1992).

Disease is initiated by attachment to ciliated epithelia (Zielinski and Ross, 1993). This results in ciliostasis, clumping and loss of cilia (DeBey and Ross, 1994), and finally the loss of epithelial and globlet cells (Debey et al., 1992). Interest in adherence mechanisms led to the preliminary characterization of a cilium binding mechanism and cellular receptor (Zhang et al., 1994a,b), and eventually to the identification of monoclonal antibodies (F1B6 and F2G5) capable of significantly blocking binding to purified cilia (Zhang et al., 1995). These monoclonal antibodies recognized a series of proteins by immunoblot; the largest protein was 97 kDa in size, now referred to as P97. Construction of a Lambda library using a novel opal suppressor strain of *Escherichia coli* (Minion et al., 1995) led to the discovery and characterization of the structural gene for P97 which codes for a 124.9 kDa protein (Hsu et al., 1997). Further analysis of the P97-containing Lambda clones demonstrated a second gene in a two gene operon now referred to as P102 (Hsu and Minion, 1998b). Genetic analysis of the P97 gene was used to identify the portion of the gene responsible for binding to cilia (Hsu and Minion, 1998a; Minion et al., 2000). The genome sequence of *M. hyopneumoniae* strain 232 revealed a series of P97 and P102 paralogs (Minion et al., 2004). These paralogs have been implicated in adherence to extracellular matrix proteins by a series of studies conducted by Steven Djordjevic’s laboratory (Burnett et al., 2006; Jenkins et al., 2006; Wilton et al., 2009; Deutscher et al., 2010, 2012; Seymour et al., 2010, 2011, 2012; Bogema et al., 2011, 2012). The participation of these paralogs in adherence to host proteins commonly found on mucosal surfaces explains why the monoclonal antibody F1B6 failed to completely block adherence to purified cilia (Zhang et al., 1995) but leaves open the question of the contribution of P97 to colonization and the disease process.

In this study, we sought to construct mutants of P97 that produced little to no functional protein and determine whether recombination could be used to construct these mutants in a site-specific manner. What is typically done with mycoplasmas is to construct a transposon library and then screen that library for a specific mutant (Hudson et al., 2006; Maglennon et al., 2013a). For genes that have a known function with a phenotype easily measured in a high throughput manner, this is a reasonable approach. For genes of unknown function or those difficult to screen for in a library of hundreds or thousands of mutants, this can be problematic. Since no studies of recombination have been reported in *M. hyopneumoniae* and transformation is difficult in this species as demonstrated with the use of transposons (Maglennon et al., 2013a), we designed an approach that offered the highest probability of success. This included optimization of *M. hyopneumoniae* growth and transformation procedures, and use of plasmid forms that should optimize recombination. Complicating these studies were the presence of membrane nucleases that could damage transforming DNA potentially reducing or eliminating transformation and or recombination (Minion et al., 1993; Henthorn et al., 2018).

This study describes the construction of P97 null mutants in *M. hyopneumoniae* by recombination and their preliminary characterization to confirm loss of protein production of P97 by immunoblot analysis, loss of function by measuring binding to purified cilia and PK15 cells, and confirmation of the nature of the recombination event by PCR. Our analyses confirm that P97 is the major contributor of adherence activity in *M. hyopneumoniae*.

## Materials and Methods

### Strains and Culture Conditions

*Mycoplasma hyopneumoniae* strain 232 was originally isolated from a pig infected with strain 11 (Mare and Switzer, 1965) and has been used to study vaccination regimens and virulence in the United States. It was grown at 37°C to mid-log phase in Friis media supplemented with 20% swine sera, 5% fresh yeast extract, 0.5% dextrose, and 2.5 μg of Cefobid (Pfizer, Inc., New York, NY, United States) per ml. For solid agar, a 2X base was prepared, filter sterilized, and added to an equal volume of agar [4 *g* Purified Agar (Oxoid #LP0028, Thermo Fisher Scientific), 0.04 *g* DEAE-dextran in 200 ml water and autoclaved to sterilize] aliquoted in 50 ml volumes for convenience. In some instances, Mycoplasma agar and Supplement from Mycoplasma Experience (Bletchingley, United Kingdom) was used according to their directions. The growth of *M. hyopneumoniae* on these two media were essentially the same as determined by colony size. Inoculated plates were wrapped in 3M Micropore tape (3M, Neuss, Germany) and incubated in a humidified incubator at 37°C in 5% CO_2_ for 18–21 days.

*Escherichia coli* were grown in Luria-Bertani broth and on agar plates supplemented with ampicillin (100 μg/ml) as needed at 37°C. The normal cloning host for plasmids was DH5α (Table 1). To generate single-stranded DNA (ssDNA), strain XL1-Blue MRF′ was used. The *E. coli* prototroph χ289 was used as a source of DNA for amplification of the wildtype *recA* gene.

**TABLE 1:** Bacteria and plasmids.

Strain or plasmid	Relevant properties	Source or reference
Bacteria		
***Escherichia coli***		
DH5α	Φ80d*lacZΔ*Ml5 *endA1 recA1 hsd*R17*(r_*k*_^–^m_*k*_^+^) sup*E44 *thi-*1 *gyr*A *rel*A1 F^–^ *Δ(lacZYA argF)*U169	BRL
XL1-Blue MRF′	*Δ(mccrA)183 Δ(mcrCB-hsdSMR-mrr)173 rec*A1 *lac^–^ end*A1 *gyr*A96 *thi-1 sup*E44 *rel*A1 [F′*pro*AB *lac*I^*q*^ *lacZΔ*M15 Tn*10* Tet^*r*]^	Stratagene
***Mycoplasma hyopneumoniae***		
232	Wild type, virulent, derived from strain 11	
232:pISM625.1 Ω1-n(ds, ss, lds)	Recombinants (dsDNA, ssDNA and Linear DNA) with pISM625	This study
232:pISM626.1 Ω1-n(ds, ss, lds)	Recombinants (dsDNA, ssDNA and Linear DNA) with pISM626	This study
**Plasmids**		
pISM603	Synthetic spiralin promoter with puromycin marker, Ap^*r*^	GeneArt
pISM641	Synthetic spiralin promoter with *E. coli recA* gene	IDT
pISM624	pSK(−) with one kb regions of P97 operon, Ap^*r*^	This study
pISM625	pISM624 with *tetM* cloned into *Eco*RI site, Ap^*r*^	This study
pISM626	pISM625 with Ps:*recA* cloned into the *Bam*HI site, Ap^*r*^	This study
pMHC9-1	Modified Himar1 transposase with hyperactive transposase and *tetM* marker	Maglennon et al., 2013a

### Plasmid Constructions

**FIGURE 1:**
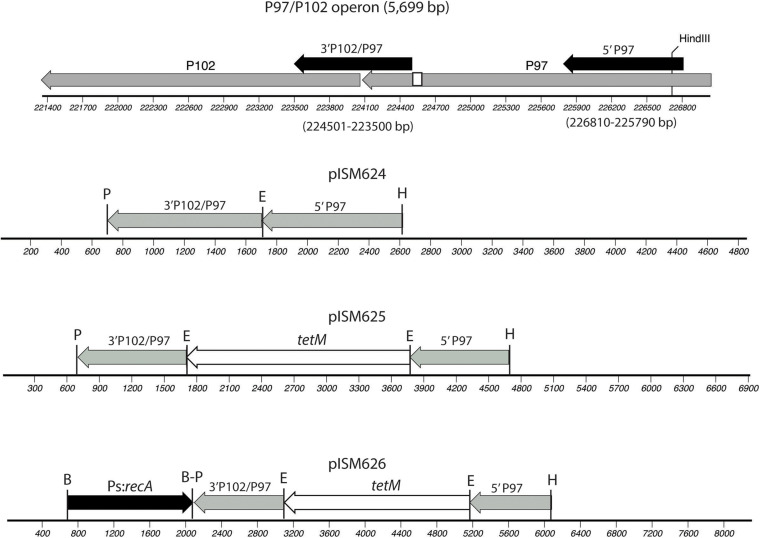
Construction of pISM624-626. The regions cloned into pISM624 are shown above the operon along with the base pair locations in the strain 232 chromosome. These fragments were amplified by PCR. The small white box in the *C*-terminal region of P97 represents the R1 region responsible for cilium binding. Note the *Hin*dIII site in the *N*-terminal P97 fragment used for cloning. The other restriction sites were added to the primers.

Plasmids were constructed on the pSK(−) Bluescript backbone (Stratagene). One kb regions of the P97 operon were amplified from strain 232 chromosomal DNA by PCR and cloned into the cloning vector using primers 5-P97-F and 5-P97-R for the *N*-terminus region in P97 and primers 3-P97-F and 3-P97-R2 for the *C*-terminus of P97 and *N*-terminus of P102 as shown in Figure 1 and described in Table 2. All PCR reactions contained 10 pmol of each primer, 1 ng template, 3 mM Mg^2+^, 0.2 mM dNTP mix, and 1 unit of Recombinant Taq DNA polymerase (Invitrogen) in 1X buffer. Cycle conditions started with 5 min 95°C denature followed by the denature, annealing, and elongation cycles (Supplementary Table 1) run on a BioRad CFX96 cycler. The downstream fragment began just beyond the R1 region responsible for cilia binding and extended into P102 (Minion et al., 2000). The primers introduced unique *Pst*I and *Eco*RI sites as shown in Figure 1. The *Hin*dIII site used for cloning was part of the P97 *N*-terminus fragment. The PCR fragments were purified, digested with *Eco*RI and then ligated together. The ligation mixture was then subjected to an additional round of PCR using primers 5-P97-R and 3-P97-R2. The resulting fragment was digested with *Pst*I and *Hin*dIII and cloned into *Pst*I-*Hin*dIII digested, dephosphorylated pSK(−) to generate plasmid pISM624. The *tetM* tetracycline resistance marker was removed from mini-Tn*4001tet* (Pour-El et al., 2002) by *Eco*RI digestion followed by purification of the fragment by agarose gel electrophoresis. This fragment was inserted into *Eco*RI-digested, dephosphorylated pISM624 to generate pISM625. To generate pISM626, the *E. coli recA* gene with a spiralin promoter was constructed as follows. A synthetic construct (GeneArt, Thermo Fisher Scientific) containing the spiralin promoter controlling the puromycin resistance marker (Algire et al., 2009) served as the template for amplification of the spiralin promoter using primers Bam_Ps_F and Ps_nde_R. The *recA* gene with a spiralin promoter was obtained by digesting pISM641 containing a synthetic Ps:*recA* sequence with *Bam*HI, isolating the Ps:*recA* fragment on an E-Gel (Invitrogen) and cloning it into the *Bam*HI site of pISM625 as described above forming pISM626 (Figure 1).

**TABLE 2:** Primers.

Primers	RE site	Sequence*
5-P97-F	*Eco*RI	AAAGGCCCCGAATTCATAGGCATAATTTCC
5-P97-R	–	AAGCAGCCAAAAAGATCCCTCAACTCTAAGAGC
3-P97-F	*Eco*RI	TCCGAATTCAAAGTTAGATTTGCTCTTGAAAG
3-P97-R2	*Pst*I	ATACTGCAGAACTTAGCCACCCAATGATGACCG
P102RT-r2		CCTTTTCCTTTTCTATCGGC
tetM-X-R		TTGTACCTTTGTCCACGCTTCC
tetM-2X-F		TGCGCCACAGGAATATCTTTCAC
bla_FWD		CTCACCGGCTCCAGATTTAT
bla_REV		CGGATGGCATGACAGTAAGA
tetM_FWD		CCATAGCGTATCCCTTCCATAAC
tetM_REV		CGCACCCTCTACTACAAACAA

*Relevant restriction sites (RE) are underlined.

### DNA Manipulations

Plasmid DNAs were purified from *E. coli* using E.Z.N.A. Plasmid DNA Mini or Midi kits (Omega Bio-Tek, Inc., Norcross, GA, United States) and quantitated using a Qubit fluorometer with the dsDNA Assay Kit (Invitrogen). Linear DNA was obtained from plasmid DNA by digestion of 10 μg of plasmid DNA with *Sal*I followed by ethanol precipitation and dissolving in water. Zymoclean Gel DNA Recovery kits were used to purify DNA fragments from agarose gels (Zymo Research, Irvine, CA, United States). PureLink PCR Micro kits (Invitrogen) were used to purify PCR fragments from PCR reactions. Chromosomal DNAs were extracted using phenol-chloroform and precipitated with ethanol (Madsen et al., 2007).

Single-stranded DNA was prepared by first transforming the XL1-Blue MRF′ strain with pISM625 and pISM626. The VCSM13 helper phage was used to infect the culture (Agilent, Santa Clara, CA, United States). A 25 ml volume of 2X YT was inoculated with a single colony of host and grown at 37°C for 1−2 h. The VCSM13 helper phage was added at 10^7^−10^8^ pfu/ml (MOI∼10) and the culture was shaken vigorously at 37°C for 1−2 h. Kanamycin was then added to 70 μg/ml to select for infected cells and the culture continued to shake overnight to reach saturation. The culture was centrifuged at 10,000 × *g* for 10-15 min to remove lysed cells and cellular debris. To each ml of supernatant, 150 μl of a solution containing 20% PEG 8000 and 2.5 M NaCl was added. The phage were allowed to precipitate for at least 1 h on ice or overnight at 4°C. The supernatant was then centrifuged at 10,000 × *g* for 10 min, the supernatant was removed and the tube recentrifuged briefly to remove any residual liquid. The pellet was resuspended in 0.5 ml of 0.3 M sodium acetate − 1 mM EDTA, pH 6.0, by vortexing. The suspension was extracted twice with equal volumes of phenol-chloroform followed by precipitation with two volumes of 100% ethanol. Following centrifugation, the final pellet was dissolved in water and quantitated on a Qubit fluorometer with a ssDNA Assay Kit (Invitrogen).

### Transformation

Mycoplasmas were transformed by electroporation using a BioRad Micropulser with the settings for bacteria and 2.5 kV in 2 mm cuvettes (Thermo Fisher Scientific). Cells were prepared as described (Pour-El et al., 2002) by centrifuging a mid-log phase culture for 20 min at 10,000 × *g*, washing three times with cold electroporation buffer (8 mM HEPES-272 mM sucrose, pH 7.4) and resuspending in the same buffer at 1:10 volume of the original culture. The suspension was placed on ice and incubated for 30 min. 100 μl of cells were incubated with 10 μg DNA dissolved in water for 30 min on ice and then transferred to a prechilled electroporation cuvette (Thermo Fisher Scientific). Following electroporation, 900 μl complete media was added, and the cuvettes were placed on ice for an additional 5 min. Using a transfer pipette, cultures were transferred to a new 1.5 mL microfuge tube and incubated at 37°C for 3 h. Cultures were spun at 10,000 × *g* for 1 min. Without disturbing the pellet, 800 μl was removed and each pellet was resuspended in the remaining fluid (∼100 μl). The cell suspension was plated onto solid Friis media plates containing 0.2 μg/ml tetracycline, wrapped with 3M Micropore tape and incubated at 37°C, 5% CO_2_ for 14-21 days. Colonies were counted and picked on a dissecting microscope with sterile glass Pasteur pipettes. The agar plug was dispensed into 1 ml broth containing 0.5 μg/ml tetracycline in a 75 × 150 mm sterile polystyrene tube, vortexed for 30 s and then the tube was placed in a 37°C incubator until a color change indicated growth (∼3 days) at which time four ml of selective media was added. Incubation continued for another 3 days when additional media was added. Final cultures were stored in aliquots at −70°C. Ten ml of grown culture was centrifuged at 6,000 × *g* for 40 min, the cells were washed twice with PBS by centrifugation in a microfuge and protein concentration was determined on the final pellet suspended in 50 μl of water for immunoblot analysis.

### Characterization of *M. hyopneumoniae* Transformants

#### Immunoblot Analysis

Twenty-five ml volumes of culture for each transformant to be analyzed were grown in Friis broth. Cells were pelleted by centrifugation (10,000 × *g* for 20 min, 4°C) and washed twice with phosphate buffered saline (PBS). The final cell pellet was resuspended in water at a 1:100 volume and protein determined by Qubit and adjusted to 1 mg/ml with water. An equal volume of 2X Laemmli Sample Buffer (BioRad, Hercules, CA, United States) was added, the samples were boiled for 5 min and centrifuged in a microfuge for 3 min prior to loading on 8–16% Criterion TGX Protein gels (BioRad). Proteins were transferred to nitrocellulose using a BioRad Criterion^TM^ blotter. Blots were blocked with 2% fish gelatin (Sigma Chemical Co.) in TS buffer (10 mM Tris–150 mM NaCl, pH 7.2) for 2 h at room temperature and then reacted with a 1:500 dilution of monoclonal F1B6 (Zhang et al., 1995) in TST buffer (TS – 0.01% Tween 20) overnight at 4°C. The blots were washed with TST and then reacted with a 1:500 dilution of alkaline phosphate conjugated goat anti-mouse IgG, IgM (H + L; Invitrogen) in TST for 2 h, washed three times and developed with SIGMA*FAST* Fast Red TR/Naphthol substrate (Sigma Chemical Co.).

#### Cilia Binding Assay

The swine cilia binding assay for *M. hyopneumoniae* has been described (Zhang et al., 1994b). The assay was modified to address the cilia binding of P97 mutants. Each mutant and strain 232 wildtype were grown in complete Friis broth to mid to late log phase, pelleted by centrifugation and washed once with PBS. Each strain was resuspended in PBS to approximately 0.5 mg/ml protein.

The assay was performed on Immulon 4 plates (Dynatech Industries, Inc., McLean, VA, United States) by coating wells with purified swine cilia solubilized with 1 mg of sodium dodecyl sulfate per mg cilia protein and diluted with sodium carbonate buffer (0.1 M; pH 9.5) to a final concentration of 10 μg protein per ml. Swine cilia were prepared as described previously (Zhang et al., 1994b) from specific-pathogen-free pigs shown to be free of *M. hyopneumoniae*. Plates were coated with 100 μl of the solubilized swine cilia preparation and incubated overnight at room temperature. The plates were then stored with coating buffer at −70°C until use. Prior to blocking, plates were thawed at room temperature, washed three times with PBS, and 200 μl per well of a 2% solution of fish gelatin in PBS was added and incubated for 2 h at 37°C to block the wells.

For the assay, the mycoplasmas representing wildtype and one each of the plasmid DNA (dsDNA), ssDNA, and linear dsDNA mutants were pelleted by centrifugation, washed once with PBS and then resuspended to approximately 1 mg/ml protein in PBS. They were added to triplicate wells, 100 μl per well, and incubated for 2 h at 37°C. Plates were incubated with R254 rabbit hyperimmune anti-*M. hyopneumoniae* strain 11 antisera (Petersen et al., 2016) diluted 1:200 in PBS for 2 h at 37°C followed by alkaline phosphatase-conjugated donkey anti-Rabbit IgG (Jackson ImmunoResearch Laboratories, Inc., West Grove, PA, United States) for 2 h at 37°C. Between incubations, the wells were washed with PBS three times, 15 min per wash. Wells were developed with 4-nitrophenyl phosphate in diethlyamine buffer (Sigma Chemical Co.) and read on a SpectraMax model 190 Molecular Devices ELISA reader at 405 nm. Wells devoid of mycoplasmas were subtracted as background controls.

#### Adherence Assay

PK15 cells were removed from liquid nitrogen storage and the cells washed and resuspended in Dulbeccos’s Modified Eagle Medium (DMEM) media (Corning Cellgro, Corning, NY, United States) supplemented with 10% fetal calf serum, 1X GlutaMAX (Gibco, Thermo Fisher Scientific), 25 mM HEPES and 25 μg/ml Cefobid, and grown at 37°C and 5% CO_2_. Confluent growth was observed in 72 h. Cells were passed by trypsin treatment and then seeded to 12 well Costar tissue culture plates (Corning) at a concentration of 1 × 10^5^ cells/well (Raymond et al., 2018). *M. hyopneumoniae* 232 and each recombinant were grown for 70 h in Friis media. Mycoplasmas were pelleted and resuspended in infection media consisting of DMEM media containing 5% fetal calf serum, 1X GlutaMAX (Gibco, ThermoFisher Scientific) and 25 mM HEPES. Dilutions were plated on Friis agar plates to determine the multiplicity of infection. Mycoplasmas were added (30 μl) to triplicate wells at a MOI of approximately 1. Mycoplasmas were allowed to adhere over night at 37°C, 5% CO_2_. Post incubation, plates were washed twice with 1 ml 1X PBS and then 350 μl trypsin was added to each well and incubated at room temperature for 10 min. The trypsinized cells and mycoplasmas were diluted 10^–1^ and 10^–2^ in Friis media, and 10 μl dilutions were spotted to Friis agar plates to determine colony forming units. Plates were incubated for 21 days at 37°C and 5% CO_2_. Colony counts were recorded and analyzed.

#### Analysis of P97/P102 Recombinants Genetic Structure

The structure of the recombination event in randomly selected mycoplasma transformants was analyzed by PCR. The PCR reactions tested for the presence of the ampicillin marker (*bla*), the *tetM* marker and two internal fragments that were diagnostic for a single cross-over event at one of two positions. Chromosomal DNAs were obtained by pelleting the cells from 5 mls of late log phase growth in broth and washing the cells three times with PBS by centrifugation. The final pellet was resuspended in PBS and 0.01% sodium dodecyl sulfate was added along with 1 mg/ml proteinase K and incubated overnight at 55°C. The solution was then extracted three times with chloroform-phenol. The resulting purified DNA was used in PCR reactions with primers for the ampicillin marker (*bla*, primers bla_FWD and bla_REV), tetM (tetM_FWD and tetM_REV), and two sets of primers to detect specific fragments of the recombination event depending on the orientation (P102RT-r2 and tetM-2X-F; tetM-X-R and 5-P97-F; Figure 5).

### Statistical Analyses

Linear regression models and analysis of variance models were run using Proc GLM ANOVA. Box plots were generated using BoXPlotR^1^.

## Results

### Plasmid Constructions

Figure 1 illustrates the plasmids constructed for this study. Regions of the P97 operon cloned included the *N*-terminal region of P97 and the *C*-terminal region of P97 downstream of the R1 repeat and extending into P102. Each fragment was obtained by PCR, digested with *Eco*RI, ligated and reamplified by PCR with the outer primers, digested with *Pst*I and *Hin*dIII and cloned into *Pst*I-*Hin*dIII digested, dephosphorylated pSK(−) vector. The resulting plasmid, pISM624, was sequenced and further modified by cloning in the *tetM* marker with its native promoter from Tn*916* at the *Eco*RI site to generate pISM625. To test for the need for RecA, the *E. coli* with a spiralin promoter gene was obtained from pISM641 and cloned into the *Bam*HI site of the MCS of pISM625 to form pISM625. Figure 1 shows relevant restriction sites, the structure of the P97 operon, and the sequences cloned.

### Transformation

The antibiotic sensitivities of *M. hyopneumoniae* 232 was determined by plating 100 μl of mid-log culture on an 85 mm petri dish containing Friis agar with various concentrations of tetracycline. This strain was sensitive to tetracycline at 0.2 μg/ml, and thus this concentration was used throughout the study to select for transformants. Transformation was accomplished by electroporation essentially as described above with a cell density of approximately 4 × 10^8^ mycoplasmas per ml. Table 3 lists the transformation frequencies of the different plasmids and forms of DNA. All transformation frequencies were at approximately 10^–7^ transformants per mycoplasma. There was no difference in frequency between the different plasmid forms (ds, linear, or ss). The presence of *recA* controlled by the spiralin promoter did not affect the transformation frequencies. All colonies appeared to grow at the same rate. It was not possible to discern a difference in growth in broth.

**TABLE 3:** Transformation frequencies.

Plasmid/form	# colonies	Tf*
**pISM625**		
ds	11	2.62 × 10^–7^
linear	11	2.62 × 10^–7^
ss	13	3.10 × 10^–7^
**pISM626**		
ds	12	2.85 × 10^–7^
linear	10	2.38 × 10^–7^
ss	13	3.10 × 10^–7^

*Transformation frequency.

### Characterization of Transformants

#### Immunoblot Analysis

**FIGURE 2:**
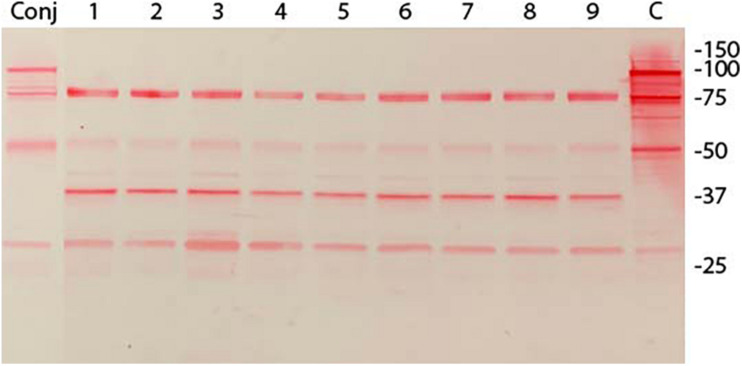
Immunoblot analysis of *M. hyopneumoniae* pISM625 transformants. *M. hyopneumoniae* strain 232 was transformed with pISM625 and pISM626. Transformants were picked, grown in Friis broth and proteins prepared for SDS-PAGE and immunoblot analysis. Transformants from pISM625 are shown above. Each lane represents an individual transformant. 10 μg of protein was loaded per lane. Molecular weight markers are on the right. Conj = conjugate only, no primary antibody; lanes 1−3, dsDNA transformants 232:pISM625.1 Ω1–3(ds); lanes 4−6, ssDNA transformants 232:pISM625.1 Ω1-3(ss); lanes 7−9, linear dsDNA transformants 232:pISM625.1 Ω1–3(lds); C = control wildtype *M. hyopneumoniae*. Blot was developed with monoclonal F1B6, which binds to the R1 repeat sequence in P97 and blocks cilia binding.

Immunoblot analysis of three randomly selected pISM625 transformants from the dsDNA, ssDNA, and linear dsDNA transformation is shown in Figure 2. The pISM626 transformants gave identical patterns by immunoblot and is not shown. The upper panel was developed with monoclonal antibody F1B6 shown to bind to the R1 region of P97 and block significant adherence to swine cilia (Zhang et al., 1995; Hsu and Minion, 1998a). The characteristic 97 kDa protein is missing from all transformants along with a number of smaller proteolytic fragments as compared to wildtype (Djordjevic et al., 2004). Remaining bands at 27, 50, and 76 kDa that can be ascribed to the conjugate cross reactivity. Additional bands at 37 kDa and a single band in the doublet at 78 kDa is thought to be due to one of the P97 paralogs, mhp385, that has four tandem R1 repeats (Deutscher et al., 2012), sufficient for F1B6 recognition (Minion et al., 2000; Petersen et al., 2019). The upper band is approximately 80–85 kDa and could represent a proteolytic fragment of mhp385 (Deutscher et al., 2012). The origin of the 37 kDa protein is unknown. It is absent in the wildtype lane, and is thought to be a product of the recombination event.

#### Cilia Binding Assay

**FIGURE 3:**
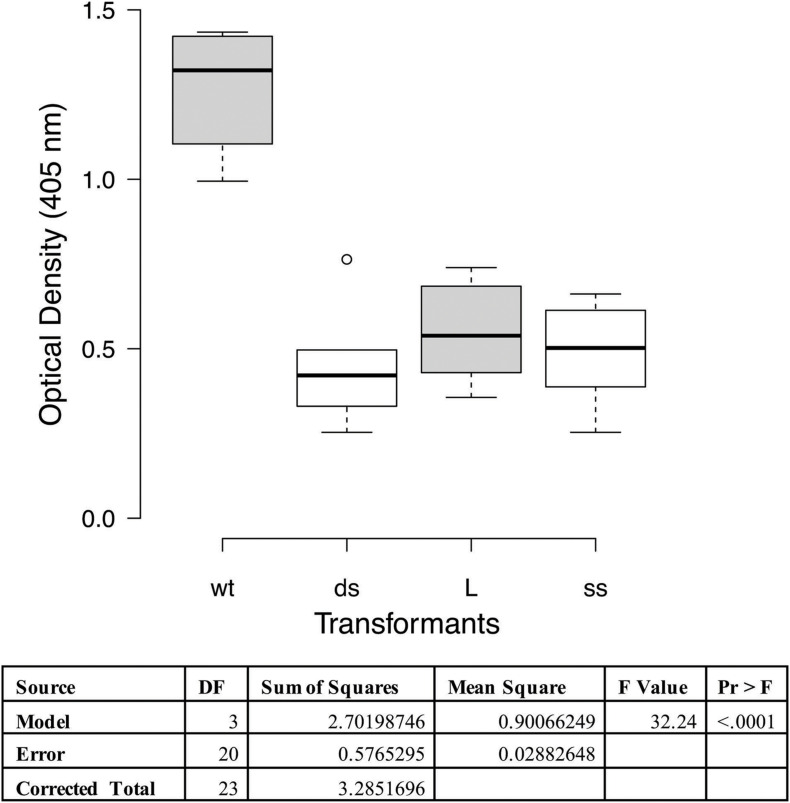
Cilia binding assay with *M. hyopneumoniae* recombinants. The data represent duplicate assays with triplicate wells per assay and are expressed as optical density at 405 nm. Proc GLM ANOVA was performed on the data. Box plots were generated using BoXPlotR (http://shiny.chemgrid.org/boxplotr/). Wt = wildtype control; ds = dsDNA transformant; L = linear dsDNA transformant; and ss = ssDNA transformant. Center lines show the medians; box limits indicate the 25th and 75th percentiles as determined by R software; whiskers extend 1.5 times the interquartile range from the 25th and 75th percentiles, outliers are represented by dots *n* = 6 sample points. Letters indicate values that are statistically different. The three transformant types are significantly different from wildtype (*p* < 0.0001).

A cilia binding assay was performed to functionally characterize the adherence activity of the P97 null mutants. In this assay one of each of the different types of transformants was tested on purified cilia and compared to the wildtype strain. Statistical analysis indicated that all three transformants had decreased binding affinity to cilia than wildtype (*p* < 0.0001; Figure 3). This reduction in binding is comparable to what was observed with the monoclonal antibody, about 60% (Zhang et al., 1995). Subsequent studies have shown that other members of the P97/P102 gene families have adherence-related activities (Seymour et al., 2010, 2011; Tacchi et al., 2014), easily accounting for the remainder of the approximately 35% cilia binding activity.

#### PK15 Cell Adherence Analysis

**FIGURE 4:**
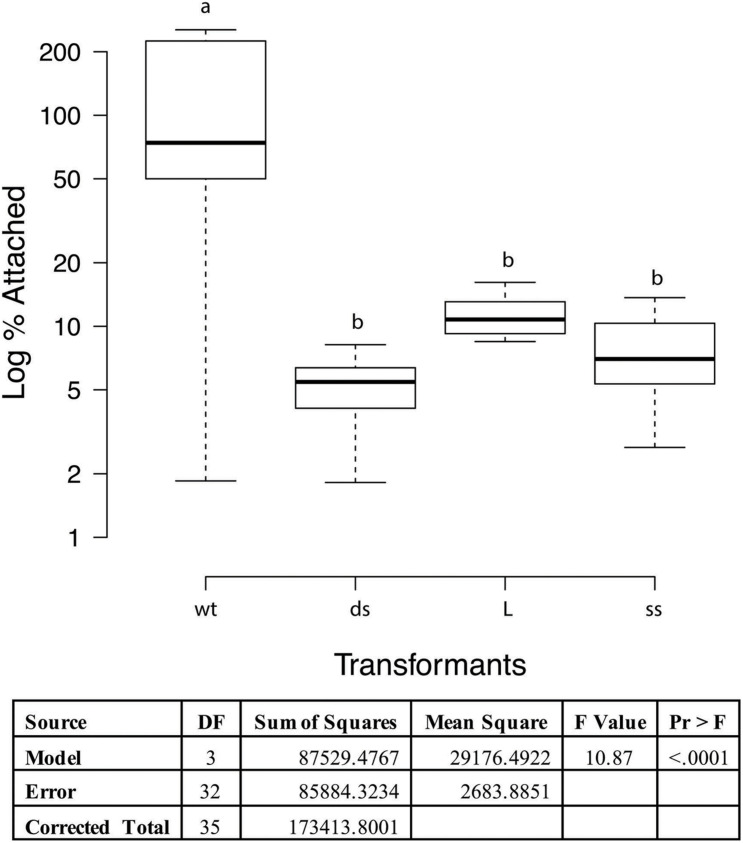
PK15 adherence assay with *M. hyopneumoniae* transformants. The data represent triplicate assays with triplicate wells for each sample and are expressed as log_10_ percent attached of the starting mycoplasma numbers. Proc GLM ANOVA was performed on the data. Box plots were generated using BoXPlotR (http://shiny.chemgrid.org/boxplotr/). Wt = wildtype control; ds = dsDNA transformant; L = linear dsDNA transformant; and ss = ssDNA transformant. The data is presented as Log_10_. Center lines show the medians; box limits indicate the 25th and 75th percentiles as determined by R software; whiskers extend 1.5 times the interquartile range from the 25th and 75th percentiles, outliers are represented by dots *n* = 9 sample points. Letters indicate values that are statistically different. The three transformant types are significantly different from wildtype (*p* < 0.0001).

Adherence to PK15 cells was analyzed to determine if loss of function occurred in recombinant strains when compared to wild type 232. When taken from liquid nitrogen storage, the PK15 cells grew readily in the DMEM supplemented media obtaining confluence in 2−3 days. Once cells were seeded to 12 well tissue culture plates, they grew to confluency overnight. Cultures of wildtype strain 232 and three mutants (one each of the different transformation sets chosen randomly) were grown to mid log phase in Friis media, pelleted by centrifugation and resuspended in infection media to approximately the same volume. Colony forming units were determined for each mycoplasma suspension and the PK15 cells were infected with 30 μl of mycoplasma suspension corresponding to approximately a MOI of 1 and incubated overnight at 37°C and 5% CO_2_. Microscopic examination of the cell layers just prior to harvesting indicated some aggregates of what appeared to be mycoplasmas primarily in the mutant containing wells (data not shown). These aggregates were not disrupted prior to plating and might result in the much of the variation seen in the assay. Since the actual numbers of wildtype and mutant mycoplasmas added to the wells varied between the cultures, the data were expressed as percentage of adherent mycoplasmas, assuming that approximately equal numbers of PK15 cells were in each well. ProcGLM and ANOVA analyses showed wild type 232 was statistically significantly different from all three recombinants (*p* < 0.0001; Figure 4).

#### Genetic Analysis of Mycoplasma Transformants

**FIGURE 5:**
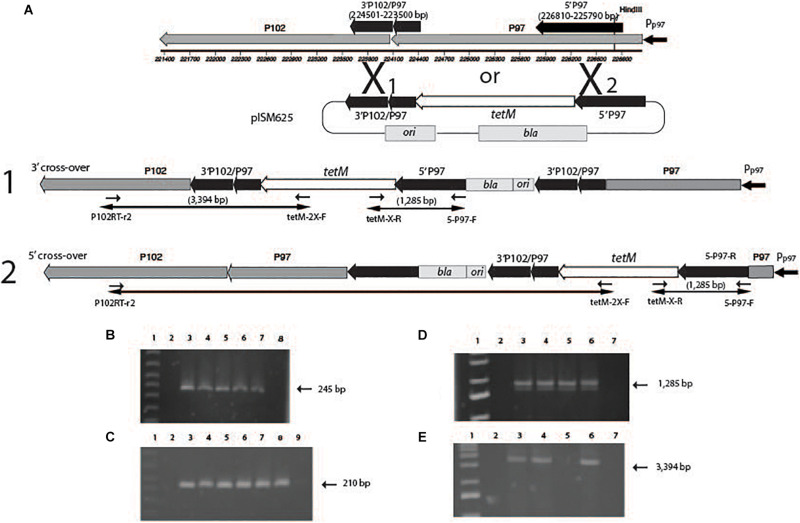
Genetic analysis of *M. hyopneumoniae* transformants with pISM625. At the top of the figure **(A)**, the P97/P102 operon is shown drawn to scale. The black arrows above the operon represent the regions cloned into pISM625 and pISM626. The large X represents potential cross-over points between the chromosome and plasmid. Single cross-over events can occur either at position X_1_ or X_2_ resulting in the corresponding structures shown in A1 and A2. **(B–D)** Agarose gels of PCR reactions with chromosomal DNAs from pISM625 transformants. Templates in all PCRs in all gels except where noted: Lanes 1, 1 kb ladder; 2, *M. hyopneumoniae* non-transformant wildtype control; 3, linear dsDNA transformant #1; 4, linear dsDNA transformant #2; 5, ssDNA transformant; 6, dsDNA transformant; 7, pISM625 positive control; and 8, water template control. In **(C)**, Lane 8, pMHC9-1 (Maglennon et al., 2013a) positive control; Lane 9, water template control. Primers used were as follows: **(B)** *bla*-specific primers (bla_FWD and bla_REV). **(C)** *tetM*-specific primers (tetM_FWD and tetM_REV). **(D)** primers tetM-2X-F and 5-P97-F. **(E)** primers P102RT-r2 and tetM-2X-F.

Figure 5 indicates the regions in the P97/P102 operon that were cloned into the recombinants. pISM625 transformants were randomly selected from dsDNA, ssDNA, and linear dsDNA transformations as described above. Analysis of those transformants by PCR is shown in Figure 5. Figure 5A describes the cloned regions of the P97 operon, the plasmid pISM625 (not drawn to scale) and the potential single cross-over recombination events that could have occurred in *M. hyopneumoniae* and the resulting genome structure. Also shown are the agarose gel results of PCR reactions (Figures 5B–D). Since all transformants were *bla* positive (Figure 5B), all transformants represent a single cross-over event with the plasmid integrating into the chromosome. Likewise, all transformants were *tetM* positive as expected since the transformants were tetracycline resistant (Figure 5C). Figure 5D is additional confirmation of the genome structure showing positive reactions with the tetM-X-R and P-P97-F primers. Figure 5E shows that three of the four transformants represent a cross-over in the #1 region while the ssDNA transformant shows a cross-over in the #2 region. The PCR product in the ssDNA transformant would have been much larger and under the PCR conditions used would not be amplified.

## Discussion

Recombination has been studied in mycoplasmas, primarily as a mechanism for antigenic variation (Burgos et al., 2012). Targeted gene knockouts have been reported for various species (Dybvig and Woodard, 1992; Cao et al., 1994; Halbedel and Stulke, 2007; Chopra-Dewasthaly et al., 2008; Allam et al., 2010; Torres-Puig et al., 2015), but not for the fastidious species *M. hyopneumoniae*. One approach that has proven useful in mycoplasmas is to construct an *oriC*-based plasmid that can replicate in the mycoplasma eventually resulting in recombination into the genome (Janis et al., 2005; Halbedel and Stulke, 2007). Another approach has been to include a *recA* gene on the transforming plasmid to enhance the frequency of recombination (Allam et al., 2010; Ishag et al., 2017). We constructed a similar plasmid in this study; *recA* from *E. coli* was fused to the spiralin promoter to control expression in the mycoplasma transformants. This promoter has been shown to function in *M. hyopneumoniae* (Maglennon et al., 2013a).

In *M. hyopneumoniae*, transformation has been a challenge, and only strain 232 has been transformed (Maglennon et al., 2013a, b). In designing a system for targeted gene knockouts in *M. hyopneumoniae*, there were several concerns. First, were the recombination systems in *M. hyopneumoniae* capable of supporting recombination? Second, would a heterologous RecA protein be necessary or contribute in a significant way? Since ssDNA is required for recombination, would ssDNA enhance integration? Finally, could a linear DNA fragment integrate into the chromosome? To test these possibilities, we constructed plasmids in the pSK(-) Bluescript backbone to allow for the generation of ssDNA by M13 infection. The multiple cloning site gave several restriction sites suitable for restriction outside the cloned sequences so that linear plasmid could be easily obtained. We chose to target the P97 gene for several reasons. First, we had considerable information regarding the function of this gene product. Second, we had immunological reagents specific for the protein that could be used for characterization of the mutants. Finally, we understood this protein to be a major virulence factor of this species and thus represent a good first target for mutagenesis.

Construction of the plasmids began by deciding on the type of recombination event we were pursuing, one that resulted in deletion of the R1 region of P97, the target for our monoclonal antibody. So we could easily screen the mutants and detect its loss by immunoblot. That dictated that gene sequences upstream and downstream of this region of P97 must be cloned along with a resistance marker that would integrate during recombination so that deletion of this region would occur. The sizes of the regions of homology were essentially arbitrary. The exact regions cloned were dictated by the presence of a *Hin*dIII site in the 5′ region of P97 for ease of cloning and designing a PCR primer that bound just downstream of the R1 region. Figure 1 shows our strategy in the cloning of the two regions. Not knowing the extent of homologous sequences needed for recombination, we chose to clone 1 kb regions of the P97 operon, which included the 5′ end of P102 as well. We also chose to use *tetM* from Tn*916* along with its native promoter for the constructs. This resistance cassette has been shown to function in *M. hyopneumoniae* (Maglennon et al., 2013a, b). To facilitate cloning, we added restriction sites to the PCR primers where needed to clone the two P97 operon fragments as shown in Figure 1. We separated the two fragments by an *Eco*RI site for the cloning of the resistance marker. In a second construct, we added *recA* from *E. coli* driven by a spiralin promoter at the unique *Bam*HI site to measure its impact on recombination frequency.

In previous studies, we were able to transform *M. hyopneumoniae* strain 232 with a modified mini-Tn*4001* by electroporation (data not shown), so we were confident in the transformation protocol. Experiments were performed with the two plasmids and three forms of DNA (dsDNA, linear dsDNA, and ssDNA) at the same time using the same lot of media. Every transformation mixture contained 10 μg of DNA independent of the form, dsDNA, ssDNA or linear. From each transformation, 5-6 out of 20-30 colonies were randomly picked for analysis. These were stored in aliquots at −70°C until use. Three transformants were chosen from each transformation for immunoblot analysis. Only transformants from pISM625 are shown in Figure 2; the nine pISM626 transformants were identical in their immunoblot patterns and are not shown. In all transformants, the characteristic banding pattern in the wildtype control reactive with monoclonal F1B6 beginning at 97 kDa with multiple additional smaller bands that result from proteolytic processing were absent. The remaining bands could be accounted for by cross reactivity with the conjugate (lane labeled Conj) and with the mhp385 protein, a P97 paralog that is processed into 88 and 27 kDa proteins (Deutscher et al., 2012). Only the 27 kDa would be detected by the monoclonal antibody because of the presence of a truncated R1 region in that portion of the protein. Another possibility is that the 75 and 37 kDa bands in the Figure 2 immunoblot represent aberrant transcription/translation activities. In *M. hyopneumoniae*, transcription can initiate intermittently even in intergenic regions (Gardner and Minion, 2010). Alternatively, these bands could be processed peptides of a larger protein. The strength of these bands on the immunoblot is not indicative of the level of expression. This monoclonal binds to a repeat region and is extremely sensitive to even low levels of proteins containing the R1 region. The exact nature of these bands can only be determined by additional studies.

The transformation frequencies in these studies were low averaging about 10^–7^ transformants per μg DNA (Table 3). Even for mycoplasmas this value is low. Interestingly, the presence of *recA* on the plasmid had no effect on this frequency. This is in contrast with previous studies (Allam et al., 2010; Ishag et al., 2017). We did not pursue whether *recA* was actually expressed since its presence in the cell is transitory; the plasmid functions as a suicide vector. Nevertheless, colonies resistant to tetracycline were obtained and all that were tested lacked P97 by immunoblot (Figure 2).

Functional analyses of the mutants by cilia binding and PK15 adherence assays confirmed our hypothesis that P97 was not properly expressed and processed in these transformants. To assess adherence activities, we used the only quantitative binding assays for *M. hyopneumoniae* along with a rabbit polyclonal antisera that would recognize many mycoplasma proteins unlike the F1B6 monoclonal antibody. The cilia binding assay was performed essentially as described by Zhang et al. (1994b). These transformants bound purified cilia significantly less well (Figure 3; *P* < 0.0001) in a manner very similar to that observed with the adherence blocking monoclonal antibody F1B6 (Zhang et al., 1995), with approximately 65−70% inhibition. All three mutants had the same level of reduced cilia adherence (Figure 3). A similar study was observed in the PK15 adherence assay. Using wildtype cells as a positive control, the percent adherence of the mutants demonstrated significantly less binding (*P* < 0.0001). This assay was performed essentially as described by Raymond et al. (2018) with modifications necessary because of the multiple strains of *M. hyopneumoniae*. Estimates of the cell density of the mycoplasma cultures had to be made prior to infection of the PK15 cells, and the final MOI was estimated to be about 1. Figure 4 shows the results of that assay. Note that the boxplot is drawn with a logarithmic scale. Each of the mutants bound poorly to the PK15 cells unlike the wildtype strain.

These studies were complicated by the presence of multiple P97 paralogs in the chromosome (Minion et al., 2004). One of the paralogs, mhp385, has a repeat region that binds the monoclonal antibody (Petersen et al., 2019) and should appear on the immunoblot as a 27 kDa processed protein. It was not easy to detect this protein in wildtype cells because of the partial proteolytic processing of P97 and its paralogs (Djordjevic et al., 2004). The paralogs also complicated the PCR analysis of the mutant genomes. By choosing primer pairs that gave negative results with wildtype DNA, we were able to determine the orientation of the recombination event without interference from the other paralogs.

In summary, recombination was used to create a P97 null mutant of *M. hyopneumoniae*. This mutation resulted in a significant reduction in its ability to bind purified cilia and adhere to PK15 cells. Future studies will involve the development of a complementation system to restore P97 function and swine infection studies with these mutants to assess the overall importance of P97 to colonization and disease.

## Data Availability Statement

The datasets generated for this study can be found in the Supplementary Data.

## Author Contributions

FM conceived the study, performed the cilia binding assay, and managed the project. JC assisted in the cloning and the *M. hyopneumoniae* transformation portion of the studies. MM performed the PK15 adherence assays and the statistical analyses. FM wrote the first draft of the manuscript. All authors contributed to manuscript revision, read and approved the submitted version.

## Conflict of Interest

The authors declare that the research was conducted in the absence of any commercial or financial relationships that could be construed as a potential conflict of interest.

## Footnotes

1
http://shiny.chemgrid.org/boxplotr/
