# Supplementary material for: Construction of Mycoplasma hyopneumoniae P97 Null Mutants
Source: Front Microbiol. 2021 Apr 22;12:518791. doi: 10.3389/fmicb.2021.518791 (PMC8101707; doi:10.3389/fmicb.2021.518791)
Supplement: Supplementary file 1 [file Table_1.DOCX]

Supplement 1.PCR conditions

| Primers | PCR Cycle Conditions* |
| --- | --- |
| 5-P97-F | (10"-95°, 30"-60°, 120"-68°) X 20, 300"-68° |
| 5-P97-R |  |
| 3-P97-F | (30"-95°, 15"-48°, 15"-68°) X 19 |
| 3-P97-R2 |  |
| tetM-2X-F | (15"-95°, 30"-48°, 210"-68°) X 29, 300"-68° |
| P102RT-r2 |  |
| tetM-X-R | (15"-95°, 30"-55°, 90"-68°) X 39, 300"-68° |
| 5-P97-R |  |
| bla_FWD | (15"-95°, 15"-51°, 30"-68°) X 34, 300"-68° |
| bla_REV |  |
| tetM_FWD | (15"-95°, 15"-52°, 60"-68°) X 25, 300"-68° |
| tetM_REV |  |

* PCR reactions contained 10 pmol each primer; 1 ng template, 1.5 mM Mg^2+^, 0.2 mM dNTP mix and 1 unit Taq DNA polymerase (Invitrogen) in 1X buffer. Cycle conditions started with a 5 min 95°C denaturation followed by the indicated cycles and ended with a 5 min final elongation step.

| § Taq DNA polymerase (Invitrogen) |
| --- |
| ¥ Q5 High-Fidelity DNA polymerase (New England Biolabs) |
